# Combining Targeted Metabolites Analysis and Transcriptomics to Reveal Chemical Composition Difference and Underlying Transcriptional Regulation in Maca (*Lepidium Meyenii* Walp.) Ecotypes

**DOI:** 10.3390/genes9070335

**Published:** 2018-07-03

**Authors:** Qiansi Chen, Meng Li, Chen Wang, Zefeng Li, Jiayang Xu, Qingxia Zheng, Pingping Liu, Huina Zhou

**Affiliations:** 1 Zhengzhou Tobacco Research Institute of CNTC, Zhengzhou 450000, China; chen_qiansi@163.com (Q.C.); wangmagic519@126.com (C.W.); ibi.zefeng@gmail.com (Z.L.); jiayangxu@126.com (J.X.); zhengqingxia916@126.com (Q.Z.); 2 College of Life Science and Technology, Central South University of Forestry and Technology, Changsha 410004, China; limeng0422@foxmail.com

**Keywords:** transcriptome, metabolome, glucosinolate, hypocotyl, Maca

## Abstract

Maca (*Lepidium meyenii* Walp.) is a traditional Andean crop with great potential for various sanitarian and medical functions, which is attracting increased research attention. The majority of previous Maca studies were focused on biochemistry and pharmacodynamics, while the genetic basis of its unique characteristics lagged due to a lack of genome information. The authors perform gas chromatography-mass spectrometry (GC/MS) analysis in the hypocotyls of three Maca ecotypes and identify 79 compounds. Among them, 62 compounds have distinct profiles among Maca ecotypes. To reveal the underlying regulatory mechanism of the chemical composition differences, de novo transcriptome sequencing is performed and the transcription profiles of three Maca ecotypes are comparatively analyzed. Functional analysis indicates several key pathways, including “starch and sucrose metabolism,” “phenylpropanoid biosynthesis,” “phenylalanine metabolism” and “plant-pathogen interaction,” are involved in regulating the chemical compositions of Maca. Combining metabolomics and transcriptomics analysis indicates transcription factors such as MYB and WRKY and mediators such as protein kinase and bifunctional inhibitors might be critical regulators of chemical composition in Maca. The transcriptome reference genome and differentially expressed genes (DEGs) obtained in this study might serve as an initial step to illustrate the genetic differences in nutrient component, secondary metabolites content, medicinal function and stress resistance in Maca.

## 1. Introduction

*Lepidium meyenii* Walp., commonly known as Maca, belongs to the Brassicaceae family. It is a traditional Andean crop that grows at altitudes between 3500 and 4500 m above sea level, in the central region of Peru [1]. Maca is also widely known as “Peruvian Ginseng,” and is prized for its nutrient component specificity, which gives it a long history of cultivation and consumption in Peru [2]. Since the 1980s, Maca has been recommended by the Food and Agriculture Organization of the United Nations (FAO) as a “neglected crop” that could be used to address the problems in human malnutrition [3]. During the 1990s, to meet the increasing market demand for Maca production, studies of Maca cultivation had been initiated in the United States, Germany, Japan, Australia and China [4]. Recently, for example, from 2001 to 2010 the exportation of Maca in Peru had increased from $1,415,000 to $6,170,000, indicating a great market potential for this plant [5]. 

The modified hypocotyl is the main edible part of Maca. The chemical composition and nutritional value of Maca hypocotyls have been investigated extensively. Compared to other tuber plants such as potatoes and carrots, Maca has a higher content of protein, fiber and unsaturated fatty acid. The amino acid composition of Maca has an excellent human nutritional profile and the content of essential amino acids is higher in Maca than in potatoes or carrots [6]. The major plant natural product metabolites of Maca have also been analyzed and identified. Uridine, malic acid and its benzoyl derivative, m-methoxyglucotropaeolin and glucosinolates glucotropaeolin are known to be the main natural product constituents of Maca [7]. Additionally, 1,2-dihydro-*N*-hydroxypyridine, referred to as Macaridine, the benzylated alkamides (Macamides) and Macaenes (unsaturated fatty acids) are chemotaxonomically unique compounds of Maca [8]. 

The increasing popularity of Maca owes largely to its powerful effects on the improvement of fertility and sexual behavior. Pharmacological studies showed that Maca extracts improved sexual behavior in male rats and mice [9]; the effects of Maca extracts in increasing sperm count and improving sperm vigor were verified in rats, mice and bulls [10,11]; moreover, Maca extracts significantly reduced prostate size in rats with benign prostatic hyperplasia (BPH) induced by testosterone enanthate (TE) [12]. Further to these effects, Maca has also reported to have the effects on latent learning, antioxidant defense improvement, protection against UV radiation, cancer resistance and enhancement of immunity [5].

Different ecotypes of Maca are described according to the color of their hypocotyls. There are 13 known ecotypes of Maca in the Junin region of Peru, ranging from white to black in color [5]. According to previous reports, various Maca ecotypes have different effects. Red Maca, for instance but neither yellow nor black Maca, significantly reduced ventral prostate size in rats [13]. However, daily sperm production (DSP) and epididymal sperm count (ESC) were significantly improved in adult male rats by black and yellow Maca but not by red Maca [14]. While in long-term treatments, it indicated that black Maca was the only ecotype that enhanced DSP and increased ESC [15]. Black Maca also appeared to have more beneficial effects than yellow and red Maca on latent learning in ovariectomized mice [16]. Regarding the effect of oxidative stress resistance, red Maca showed the highest antioxidant activity, while mice treated with black Maca showed the highest superoxide dismutase (SOD) levels [17]. Furthermore, black Maca and red Maca have protective effects on bone architecture in ovariectomized rats but yellow Maca has not [18]. Varying combinations of natural products are the basis of the differing colors of the ecotypes and the differential accumulation of natural product compounds or groups of compounds might be responsible for varying efficacies as therapies. A recent study demonstrated a significant correlation between the colors of Maca hypocotyls and total content of glucosinolates (GLs), which is suggested as a chemical marker for Maca biological activity, indicating the color of hypocotyls should be an intuitive signal for the difference effects of Maca ecotypes [5,19].

As a “neglected crop” with excellent potential applications, Maca is attracting increasing research attention. However, Maca studies to date have largely focused on the biochemistry, nutriology and pharmacology levels; the genome data were available for Maca until recently [20]. To interpret the functional elements of the Maca genome and to reveal the molecular constituents of cells and tissues in different Maca ecotypes, the authors perform metabolome analysis by Gas Chromatography–Mass Spectrometry (GC/MS) to reveal the difference of compound composition among the three most widely cultivated Maca ecotypes (yellow, black and violet). Moreover, the differentially expressed genes (DEGs) among these ecotypes are analyzed and establishment of the correlation between regulation factors of DEGs and compound position difference, which should in theory represent critical information about the basis of the phenotypic, nutritional and pharmacological differences among Maca ecotypes is made.

## 2. Materials and Methods 

### 2.1. Plant Materials

All Maca plants were planted in the Maca planting center in the same field of Dahai village (2120 m altitude), Huize county, Yunnan province, China, where annual average temperature is 12.7 °C and annual rainfall is 800 to 1000 mm, in March of 2014. The hypocotyls, which were randomly collected in January of 2015, were all similar in size and were all free of disease, damage and decay. Six hypocotyls were sampled for each ecotype. The overground parts of the Maca plants were removed and the hypocotyls were quickly cleaned under running water. Then the samples were immediately frozen in liquid nitrogen and stored at −80 °C until used.

### 2.2. Sample Preparation

The freshly frozen tissues were ground to powder in liquid nitrogen, filtered using a 40-mesh sieve and stored at −80 °C until the metabolic study. The samples were passed through lyophilization prior to further treatment. The tissue powder (20 mg) was added to a 2 mL tube and soaked in 1.5 mL of an extraction solvent (containing isopropanol/acetonitrile/water (3/3/2 *v*/*v*/*v*) with 15 μL (2 mg/mL) of vanillic acid as an internal standard). All extracts were sonicated for 1 h and then centrifuged at 15,000 rpm at 4 °C for 10 min. The supernatant (1.3 mL) was transferred to a new 1.5 mL tube and then centrifuged at 15,000 rpm at 4 °C for another 10 min, when 500 μL of new supernatant was collected by a conical insert of a 2 mL glass vial and dried under nitrogen flow on an N-EVAP Nitrogen Evaporator. The silylation reaction to increase the volatility of the metabolites was conducted by adding 50 μL of *N*,*O*-Bis (trimethylsilyl) trifluoroacetamide (BSTFA) to the sample and then incubating it for 60 min at 50 °C. 

### 2.3. Gas Chromatography–Mass Spectrometry Analysis

To perform metabolomic analysis, six hypocotyls for six biological replicates per ecotype were used. GC-MS analysis was performed on an Agilent 7683B series injector (Agilent, Santa Clara, CA, USA) coupled to an Agilent 6890N series gas chromatograph system and a 5975 mass selective detector (MSD) (Agilent, Santa Clara, CA, USA). Agilent DB-5MS column (0.25 μm, 0.25 mm × 30 m, Agilent Technologies, Inc., Santa Clara, CA, USA) was applied. The column process was as follows: temperature was 70 °C for 4 min and then increased at 5 °C /min to 310 °C for 15 min. The injection temperature was set at 300 °C and the injection volume was 1 μL with a 10:1 split ratio by applying Helium (99.9995%, Keyi Gas Inc., Zhengzhou, China) as a carrier gas. The column was equipped with a linear velocity control model at 1.2 mL/min. Prior to the instrumental analysis, the mass spectrometer was tuned using perfluorotributylamine (PFTBA) to obtain optimum performance. A simultaneous full scan-selected ion monitoring mode (Scan-SIM) was used to acquire the data. The mass spectra scanning scope was set to 33–600 *m*/*z* in the full scan mode and 90 chromatographic peaks of 25 groups were set in the selected ion monitoring. The scan speed is 2.59 scan/s^−1^ and the solvent cut time is 5.0 min. The temperatures of the interface and the ion source were adjusted to 280 and 230 °C, respectively. The detector voltage was maintained at 1.2 kV and the electron impact (EI) model was selected to achieve ionization of the metabolites at 70 eV.

### 2.4. RNA Extraction, Quantification and Quality Analysis

**Figure 1:**
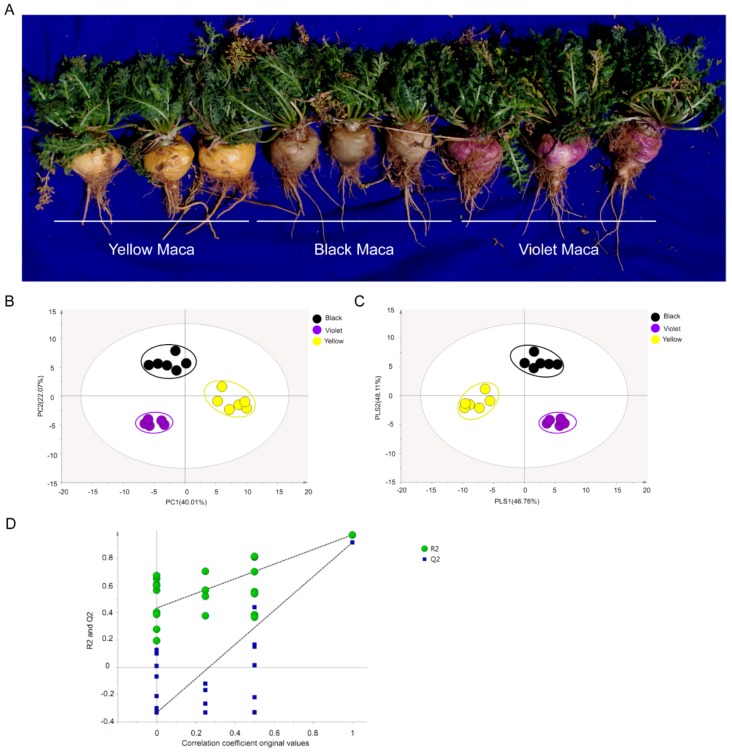
Principal Component Analysis (PCA), partial least-squares-discriminant analysis (PLS-DA) analysis and permutation tests of Maca metabolite profiling data. (**A**) Photograph shows the three different Maca ecotypes. (**B**) PCA analysis, scores plot of principal components analysis of different ecotypes of Maca. (**C**) PLS-DA score plots, based on metabolite profiling data of different ecotypes of Maca. (**D**) Permutation tests of PLS-DA models, the permutation tests were carried out with 200 random permutations. Each point represents a metabolite profile of a biological replicate.

For RNA extraction, the same six hypocotyls were sampled and randomly divided into two groups for each ecotype, representing two biological repeats. The mixed samples were named b1 and b2 (black Maca), y1 and y2 (yellow Maca) and v1 and v2 (violet Maca) (Figure 1). The total RNA of each sample was extracted using an E.Z.N.A. Plant RNA Kit (Omega, R6827-01) (Omega Bio-tek Inc., Norcross, GA, USA). Total RNA was quantified and analyzed for quality with an Agilent 2100 Bioanalyzer (Agilent Technologies). One μg of total RNA with a RNA integrity number (RIN) value above 7 was used for the subsequent preparation of libraries. 

### 2.5. Library Construction and RNA-Seq

To construct the library, the NEB Next Poly (A) mRNA Magnetic Isolation Module (New England Biolabs, Ipswich, MA, USA) was used to accomplish the poly (A) mRNA isolation, fragmentation and priming. First strand complementary DNA (cDNA) was synthesized using ProtoScript II Reverse Transcriptase (NEB) and Second Strand Synthesis Enzyme Mix (NEB) were used to synthesize first strand cDNA and second strand cDNA, respectively. AxyPrep Mag PCR Clean-up (Axygen Biosciences, Union City, CA, USA) was used to further purify Double-stranded cDNA. Then, End Prep Enzyme Mix (NEB) was applied to add adaptors. AxyPrep Mag PCR Clean-up kit (Axygen) was used to recover fragments of ~400 bp (with the approximate insert size of 250 bp). Each sample was then amplified by PCR according to instructions. The PCR products were purified and then validated using an Agilent 2100 Bioanalyzer (Agilent, Santa Clara, CA, USA) and quantified via Qubit and real time PCR analysis (Applied Biosystems, Foster City, CA, USA). Sequencing was carried out using a 2 × 125 paired-end (PE) configuration with Illumina HiSeq 2500 instrument according to the manufacturer’s instructions (Illumina, San Diego, CA, USA) The sequences were processed and analyzed with GENEWIZ software (GENEWIZ, South Plainfield, NJ, USA).

### 2.6. Sequence Analysis and De Novo Assembly

Clean data were obtained by removing reads containing adapters, reads containing ploy-N and low-quality reads from the raw data. The clean reads were assembled into contigs using the Trinity method with an optimized k-mer length of 25 for de novo assembly [21]. According to the paired-end information of the sequences, contigs were then linked into transcripts which were further clustered using a CD-Hit-EST (version 4.6.1, La Jolla, CA, USA) program with a threshold of 98% identity. The longest transcripts in the cluster units were regarded as unigenes to eliminate redundant sequences. The longest transcripts in the cluster units were regarded as unigenes to eliminate redundant sequences and then the unigenes were assembled to produce the final assembly used for annotation.

### 2.7. Functional Annotation

The final assembled unigenes were searched against the Nr (NCBI non-redundant protein sequences) database to identify putative gene functions using the BLAST algorithm [22] with an E-value cut-off of 10^−5^. Additionally, by using the Blast2GO (BioBam Bioinformatics, Valencia, Spain, version 2.3.5) program, GO (Gene Ontology) terms were extracted from the best hits obtained from BLASTx analysis against the Nr [23]. The unique sequences were further searched against the Swiss-Prot database (a manually annotated and reviewed protein sequence database), the COG database (Clusters of Orthologous Groups of proteins) and the KO database (KEGG Ortholog database) [24] (using the BLAST algorithm with E-value cut-off of 10^−5^) to predict possible functional classifications and molecular pathways.

### 2.8. Differential Gene Expression Analysis

Using Bowtie 2 (Johns Hopkins University, Baltimore, MD, USA, version 2.3.3) [25], all reads were mapped onto the nonredundant reference transcriptome to quantify the abundance of transcripts. Uni-transcript abundance differences between the samples were calculated by using the DESeq R package (1.10.1) based on the ratio of the Reads Per Kilobase per Million mapped reads (RPKM) values [26]. The P values were adjusted using a Benjamini–Hochberg approach for controlling the false discovery rate. Genes with an adjusted *p*-value < 0.05 were differentially expressed. Uni-transcripts with an absolute value of log2 ratio ≥ 2 and an FDR significance score <0.001 were selected for subsequent analyses.

### 2.9. Correlation Analysis between Metabolome and Transcriptome Data

Pearson correlation coefficients were calculated for metabolome and transcriptome data integration. The authors calculated the mean content of metabolites of Maca ecotypes and the mean value of transcript abundance of DEGs together. The coefficients were calculated from log2 (fold change) of each metabolite and log2 (fold change) of each transcript. Correlations corresponding to a coefficient with R^2^ > 0.9 were selected. The relationships between metabolome and transcriptome data were demonstrated by using Cytoscape (The Cytoscape Consortium, San Diego, CA, USA, version 2.8.2).

### 2.10. qPCR Analysis

Total RNA was reverse transcribed to generate the first strand cDNA using a PrimeScript™ RT reagent Kit with gDNA Eraser (Takara, Kyoto, Japan, RR047B). The cDNA was diluted 1:4 in distilled water and used as template for qPCR. The Maca actin gene (LmActin) was used as an internal control for normalization. The qPCR reaction was performed on an ABI 7500 qPCR instrument. Real-time PCR reactions were performed using SYBR^®^ Premix Ex Taq™ II (Tli RNase H Plus, Takara Bio, Kusatsu, Japan), ROX plus (RR82LR, TaKaRa Bio, Kusatsu, Japan). The 2^−ΔΔCT^ method [27] was used for relative expression quantitative analysis. The primer sequences used in the qPCR analysis are shown in Table S4. 

## 3. Results

### 3.1. Metabolic Differences among Three Maca Ecotypes

Gas Chromatography–Mass analysis was used to evaluate the metabolite composition in Maca hypocotyls. Comparing the mass spectra of analyte peaks with those of commercial reference standard compounds identified a total of 79 metabolites (File A1). The data were subjected to PCA (principal component analysis) and the results showed that three Maca ecotypes could be clearly separated in the PC1 × PC2 score plots (Figure 1). The first two PCs accounted for 62.08% of the total variance of the data. Then, orthogonal partial least-squares-discriminant analysis (OPLS-DA) models were generated and the results showed significant differences among Maca ecotypes (Figure 1). Furthermore, the Q2 and R2 values in the permutation test were higher than in the OPLS-DA model (Figure 1). Based on these results, the authors used the first two components to examine the metabolite profiles of Maca.

**Figure 2:**
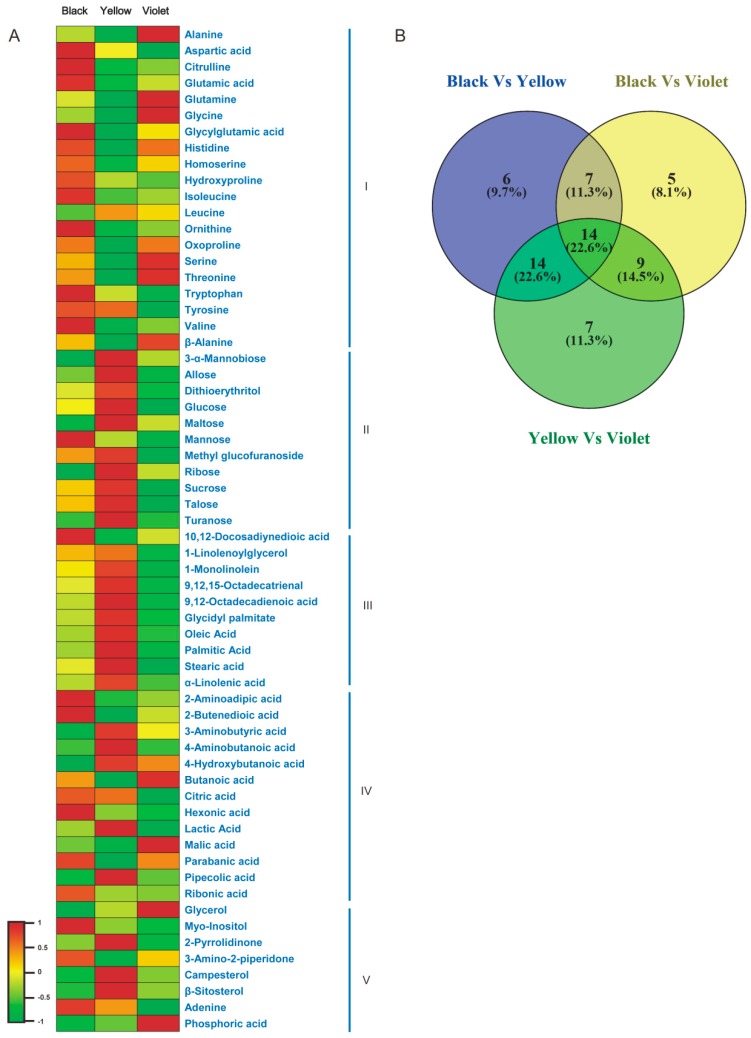
Metabolite profiles in Maca ecotypes. (**A**) The profiles of 62 metabolites in different Maca ecotypes. (**B**) The similarities and differences of metabolite composition among Maca ecotypes.

Through the combining of the VIP (variable importance in the projection) values in the loadings plot (File A2), metabolites with VIP > 1 were selected as differentially accumulated metabolites. Sixty-two compounds were identified thusly and their content was compared among Maca ecotypes (Figure 2). The results showed that the content of most amino acid compounds was significantly lower in yellow Maca than those in other ecotypes. The content of acidic amino acids (aspartic acid, glutamic acid and Glycylglutamine acid) and aromatic amino acids (tryptophan and valine), as well as derivatives of arginine (citrulline and ornithine), were highest in black Maca, while the content of hydrophilic amino acids (glycine, serine and threonine) were highest in violet Maca. Moreover, violet Maca had the highest content of alanine and β-alanine, which are essential for fertility ability and muscle development of animals. Conversely, the contents of most carbohydrate compounds were highest in yellow Maca with an exception (mannose content was highest in black Maca), as well as most fatty acid compounds (an exception was that 10,12-Docosadiynedioic acid content was highest in black Maca). Most carbohydrates and fatty acids showed relatively lower content in violet Maca. Additionally, the content of 2 sterols (campesterol and β-sitosterol) were detected most highly accumulated in yellow Maca. 

Among the 62 differentially accumulated metabolites, 14 compounds were identified to have different content in all analyzed Maca ecotypes, including six amino acids (glycine, alanine, threonine, glutamine, aspartic acid and glycylglutamic acid), four carbohydrates (glucose, ribose, mannobiose and talose), three organic acids (butanoic acid, lactic acid and 2-butenedioic acid) and one fatty acid (stearic acid) (Figure 2). The profiles of these metabolites might be candidate markers to identify different Maca ecotypes.

### 3.2. Transcriptome Sequencing and De Novo Assembly of Maca

To obtain a comprehensive Maca transcriptome, six cDNA samples prepared from mRNA of black, yellow and violet Maca hypocotyls were sequenced using the Illumina sequencing platform, respectively. Following removal of adaptor sequences and discarding low quantity reads, these samples generated 3.79 Gb (b1), 5.62 Gb (b2), 4.97 Gb (y1), 6.30 Gb (y2), 5.04 Gb (v1) and 5.13 Gb (v2) of quality transcriptome data. A summary of the sequencing output is presented in Table S1.

All high-quality reads were de novo assembled using the “Trinity” program. Further assembly was then performed with the TGICL program (The Institute of Genomic Research, Rockville, MD, USA). Redundant sequences were removed. Finally, a total of 9.16 Gb data, containing 115,866 unigenes, was obtained; this data set included all the transcripts built from high quality reads of the hypocotyls of the three Maca ecotypes. The distribution of the sequence lengths in the data set is shown in Figure S1. Using the assembled 9.16 Gb data as reference, the clean reads of six samples were mapped into the unigene library by the Bowtie 2 program. The mapping results are shown in Supplementary Table S1. 

### 3.3. Functional Annotation

All 115,866 unigenes were annotated using the BLAST algorithm (E-value < 1 × 10^−5^) against multiple protein databases, including the NCBI non-redundant protein (Nr) database [28], the UniProt/Swiss-Prot database [29], the Gene Ontology (GO) database [30], the Kyoto Encyclopedia of Genes and Genomes (KEGG) database [31] and the Cluster of Orthologous Groups of proteins (COG) database [32]. A total of 73,113 (63.10%) unigenes were thus annotated. An overall summary of the functional annotation results is depicted in Table 1. Among all annotated Maca unigenes, 63,230 (86.48%) of them had significant matches with genes from *Arabidopsis thaliana* (34,757, 47.54%) and *Arabidopsis lyrata* subsp. lyrata (28,473, 38.94%). As a member of the Brassicaceae family, it is not surprising that Maca sequences are highly homologous to the *Arabidopsis thaliana* genome. This fact should greatly facilitate future studies and crop improvement efforts concerning Maca.

Gene Ontology analysis showed that 57,591 annotated unigenes were distributed into the three categories with 43.07% of these in the biological processes category, 23.58% in the molecular function category and 33.98% in the cellular component category. The most abundant GO terms in the biological processes category were “cellular process,” “metabolic process,” and “response to stimulus.” Regarding the molecular function category, the most abundant GO terms were “binding,” “catalytic,” and “transcription regulator.” Concerning the cellular components category, the most abundant GO terms were “cell part,” “organelle,” and “organelle part” (Figure S2).

Vis a vis the COG classification, 49,846 unigenes were annotated into 24 COG categories (Figure S3). Beyond the “general function prediction” group, the second largest group was “posttranslational modification, protein turnover, chaperones,” followed by “translation, ribosomal structure and biogenesis,” “replication, recombination and repair,” “amino acid transport and metabolism,” “carbohydrate transport and metabolism,” “transcription,” and “function unknown.” The smallest groups were “cell motility,” “nuclear structure,” and “chromatin structure and dynamics.” 

Fully 8287 unigenes were assigned into 348 pathways in the KEGG analysis. The pathways involving the largest number of unigenes were, “ribosome,” followed by “carbon metabolism,” “biosynthesis of amino acids,” “spliceosome,” “protein processing in endoplasmic reticulum,” “plant hormone signal transduction,” “RNA transport,” “purine metabolism,” and “oxidative phosphorylation” (Table S3).

### 3.4. Comparison of Transcriptome Data among Three Maca Ecotypes

**Figure 3:**
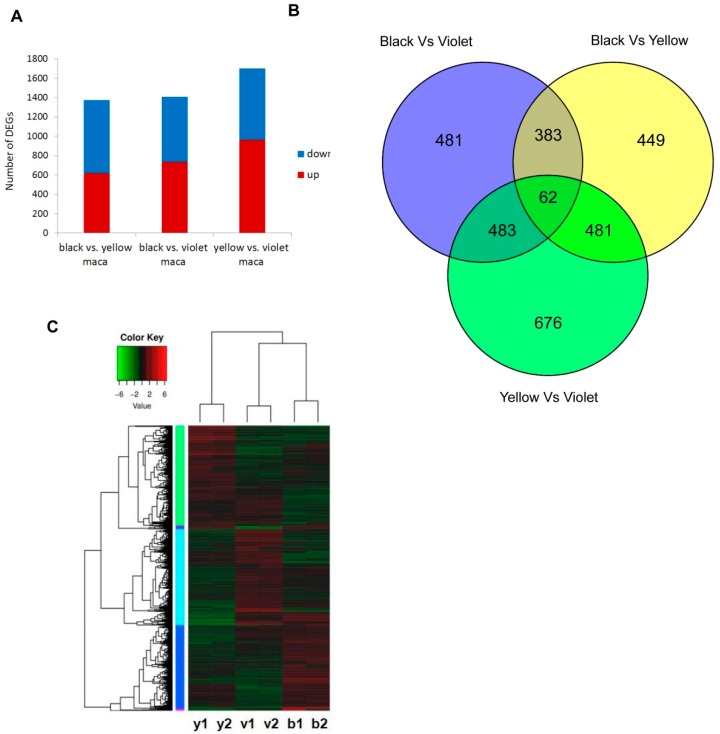
Transcription profiles in Maca ecotypes. (**A**)The number of differentially expressed genes (DEGs) among Maca ecotypes. (**B**) The similarities and differences of the number of DEGs among Maca ecotypes. (**C**) Comparison of the transcriptomes among Maca ecotypes by the heat map.

The DEGs among three Maca ecotypes were identified by DESeq analysis. Altogether, there were 1375 DEGs between black and yellow Maca, 1409 DEGs between black and violet Maca and 1702 DEGs between yellow and violet Maca, identified (Figure 3, File A3–5). Among them, 62 genes showed different expression in all Maca ecotypes (Figure 3, File A6). The results of expression profiles showed that black and violet Maca were more similar in expression profile, while yellow Maca showed a slightly different expression profile with other ecotypes (Figure 3). 

**Figure 4:**
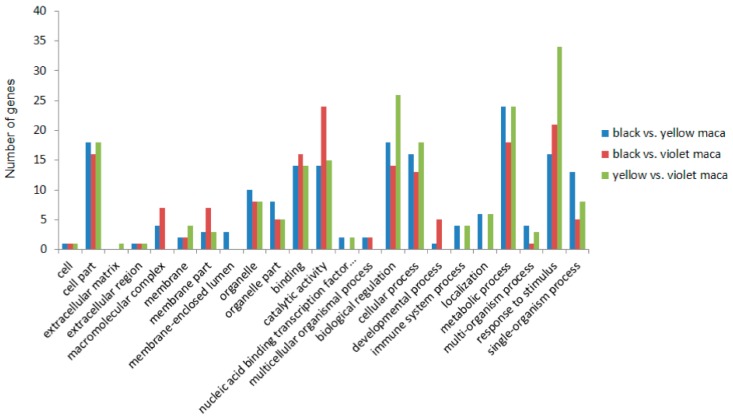
Functional annotation of DEGs based on gene ontology (GO) categorization. The DEGs were enriched in different GO terms. The GO terms such as “cell part,” “binding,” “catalytic activity,” “biological regulation,” “cellular process,” “metabolic process” and “response to stimulus,” include most highly enriched DEGs.

The identified DEGs were completed by GO analysis; the results illustrated that the most highly enriched GO terms of those DEGs were “metabolic process,” “biological regulation,” “response to stimulus,” and “catalytic activity.” DEGs between black and yellow Maca were more enriched in “single-organism process” and “organelle part” pathways than other comparisons; DEGs between black and violet Maca were more enriched in “catalytic activity” and “membrane part” pathways than other comparisons; and DEGs between yellow and violet Maca were more enriched in “response to stimulus” and “biological regulation” pathways than other comparisons (Figure 4).

Regarding the KEGG classification, it indicated that the DEGs between black and yellow Maca were enriched in the pathways of “starch and sucrose metabolism,” “phenylpropanoid biosynthesis,” “plant-pathogen interaction,” and “phenylalanine metabolism”; DEGs between black and violet Maca were enriched in the pathways of “starch and sucrose metabolism,” “plant hormone signal transduction,” “phenylpropanoid biosynthesis,” and “photosynthesis”; DEGs between yellow and violet Maca were enriched in the pathways of “starch and sucrose metabolism,” “phenylpropanoid biosynthesis,” “drug metabolism-cytochrome P450,” and “phenylalanine metabolism” (Figure S4). The expression profiles of DEGs involved in “starch and sucrose metabolism,” “phenylalanine metabolism,” “phenylpropanoid biosynthesis,” and “flavonoid biosynthesis” were analyzed in different Maca ecotypes (Figure S5). It demonstrates that violet Maca had stronger expression of genes involved in starch and sucrose metabolism than black or yellow Maca. Yellow Maca had weaker expression of genes related to phenylalanine metabolism, phenylpropanoid biosynthesis and flavonoid biosynthesis than that of black or violet Maca. 

Some genes especially expressed in one or two ecotypes were paid additional notice. Most significant genes were shown in Table 2. Among them, it is notable that the transcription of a gene encoding MYB transcription factor and a gene encoding chalcone synthase, which are involved in anthocyanin biosynthesis, were detected expressing only in violet Maca, implying their roles in violet color determination. Meanwhile, the transcriptions of two genes respectively encoding early light-induced protein and ultraviolet-B receptor were detected in black and yellow but violet Maca, indicating the UV resistance of violet Maca. The transcription of a gene encoding disease resistance-responsive, dirigent domain-containing protein were detected only in yellow Maca, which might help yellow Maca to be more resistant to disease than other ecotypes. Moreover, the transcriptions of two genes respectively encoding ubiquitin-conjugating enzyme E2 and aspartic proteinase-like protein were detected in black and violet but yellow Maca. Regarding black Maca, a gene encoding a plastidic glucose transporter, especially was expressed in it, which might cause the composition difference of glucose and sucrose. The authors also found two genes encoding retrovirus-related Pol polyprotein from transposon TNT 1-94 only expressed in yellow and violet Maca, indicating the different frequency of transposition events in black Maca. Additionally, the transcription of a gene encoding DNA ligase was detected in black Maca but no other ecotypes, suggesting the occurrence of DNA recombination in black Maca. These genes were involved in critical pathways such as sucrose and hexose transport, secondary metabolite biosynthesis, response to biotic and abiotic stress and DNA repair, which are supposed to differ in the phenotypes of Maca ecotypes. Conversely, these genes have the potential to be markers for Maca ecotype identification.

To summarize, the transcriptome data revealed some differences in the metabolism of the three Maca ecotypes. The authors note again that these differences might relate to the known differences in nutritional quality among the ecotypes. 

### 3.5. Experimental Validation of Differentially Expressed Genes

To validate the expression profiling results from the RNA-seq results, six genes, which showed quite different expression among Maca ecotypes, were selected to be analyzed by qPCR. The selected genes encoded a MYB transcription factor (c17076_g1_i1), a chlorophyll A-B binding family protein (c21560_g1_i1), an alpha/beta-Hydrolase superfamily protein (c35506_g1_i2), a phosphatidic acid phosphatase (PAP2) family protein (c40541_g1_i1), a chalcone and stilbene synthase family protein (c40810_g1_i1) and a plastidic GLC translocator (c43742_g2_i1), respectively. The expression profiles of these genes, as measured by qPCR, were compared among the three ecotypes. The qPCR and RNA-Seq results were highly similar (Figure S6). 

### 3.6. Correlation Analysis between Differentially Expressed Genes and Metabolites

**Figure 5:**
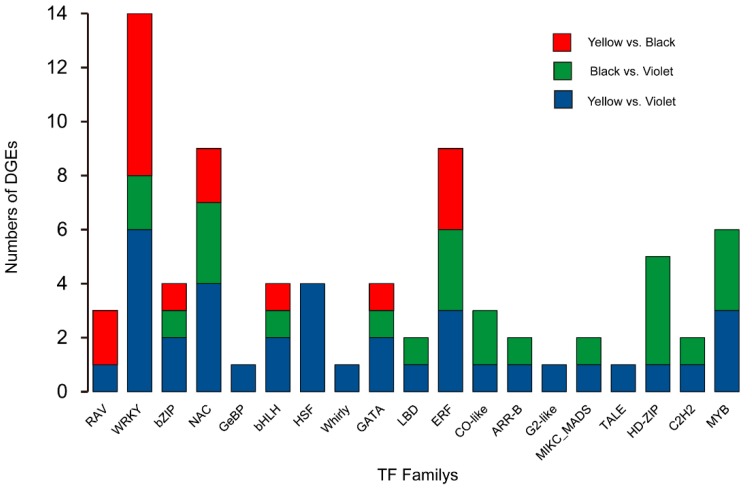
Distribution of differentially expressed transcription factors in Maca ecotypes.

To find potential regulatory factors which determine, or at least impact the metabolic difference among Maca ecotypes, the genes’ encoding transcription factors were analyzed in the sequencing data. Fully 55 genes encoding transcription factors were identified from DEGs. Among them, the *WRKY*, *ERF*, *NAC*, *MYB* and *HD-ZIP* gene family had the largest numbers involved. The authors noticed that *GeBP*, *HSF*, *Whirly*, *G2-like* and *TALE* gene were differentially expressed just between yellow and violet Maca; *MYB*, *HD-ZIP*, *LBD*, *CO-like*, *ARR-B*, *MIKC_MADS* and *C2H2* genes had no significant expression difference between yellow and black Maca and the transcript abundance of the *RAV* gene was not significantly different between black and violet Maca (Figure 5).

**Figure 6:**
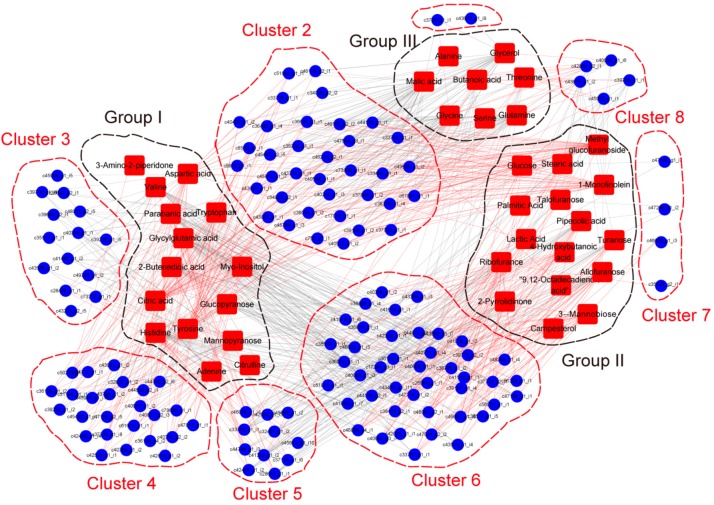
Connection network between regulatory genes and metabolites. The networks between metabolome and transcriptome data were visualized by the Cytoscape software (The Cytoscape Consortium, San Diego, CA, USA, version 2.8.2). Metabolites were divided into groups and DEGs were divided into clusters. Red lines represent positive correlations and grey lines represent negative correlations.

Furthermore, for combining transcriptomics and metabolomics analysis, identified genes encoding transcription factor, together with genes encoding mediators including kinase, protease, chaperone protein, cytochrome P450 family members and, especially, myrosinase related proteins, which are essential for glucosinolates hydrolysis, were selected from DEGs. Correlation tests were carried out between quantitative changes of the metabolites and transcript abundance of selected genes. Altogether, 140 transcripts with strong correlation coefficient values (R^2^ > 0.9) with 39 metabolites were identified. Correlation results showed 39 metabolites were divided into three groups and 140 transcripts were separated into eight clusters (Figure 6). Group I, II and III included metabolites with the highest content in black, yellow and violet Maca, respectively. Among analyzed regulatory genes, genes encoding MYB transcription factors were enriched in Cluster 2 and had a strong positive correlation with the content of malic acid and glycine, while having a negative correlation with the content of tyrosine and 1-monolinolein, uniformly. Genes encoding WRKY transcription factors were enriched in Cluster 6 and showed a positive correlation with the content of glycylglutamic acid and histidine, while a negative correlation with the content of 3-α-mannobiose and allofuranose. Gene encoding protein kinase was mainly enriched in Cluster 6 and showed a strong negative correlation with the content of lactic acid and 3-α-mannobiose. Two genes encoding bifunctional inhibitor/lipid-transfer protein were found in Cluster 5 and showed a significant positive correlation with the content of allofuranose while having a significant negative correlation with the content of histidine. A myrosinase gene in Cluster 8 showed a strong correlation with the content of allofuranose, turanose and pipecolic acid, while having a negative correlation with the content of histidine. Meanwhile, a gene encoding myrosinase binding protein was found in Cluster 3 and it showed a positive correlation with the content of adenine and tryptophan (File A7).

## 4. Discussion

Maca has been numerously documented as a promising crop with nutritious and sanitarian potential. However, the advocated multiple functions, which differ in diverse Maca ecotypes, largely determines the price of Maca products. The price of black Maca is about three times that of yellow Maca and it is widely believed that the blacker the Maca, the higher price it deserves, for example. Therefore, in a Maca market, the quality of the product is always assessed just by their appearance of color. The relationship between Maca color/ecotypes and function remain unclear and customers are often confused in this case. The authors systematically analyzed the three most common Maca ecotypes with different market prices in metabolic and transcription level, to discover different compounds, genes and underlying regulatory mechanisms among Maca ecotypes. Meanwhile, special compounds and genes found in the present study are expected to be candidate markers for identifying Maca ecotypes. 

### 4.1. Overview of Metabolome of Maca Ecotypes

Gas Chromatography–Mass analysis has identified 79 compounds in Maca hypercotyls. The compounds of different Maca ecotypes were clearly separated in Principal Component Analysis (PCA) and OPLS-DA, indicating the chemical composition difference among Maca ecotypes. Furthermore, the authors noticed that yellow Maca has obviously lower amino acids content, while significantly higher content of carbohydrates, organic acids and sterols than other ecotypes, suggesting that yellow Maca has higher soluble sugar content, higher long-chain fatty acid content and lower protein content. 

Amino acids are essential precursors of glucosinolates, which is an important active ingredient of Maca [6]. Aliphatic amino acids including Ala, Leu, Ile, Val and Met, are the precursors of methoxybenzyl glucosinolate, aromatic amino acids, including Phe and Tyr, which are the precursors of benzyl glucosinolate and Trp is the precursor of indole glucosinolate [33]. This study’s results show that black Maca has the highest content of Ile, Tyr and Trp, which is consistent with previous reports demonstrating that black Maca is highest in glucosinolate content [6,7,34]. These results indicate that yellow Maca is more suitable for providing basic material and energy as food, while black Maca is preferred to be used in health products. Regarding violet Maca, it has the medium content between black and yellow Maca of most metabolites, while it also has its specific metabolites profile. The content of hydrophilic amino acids including Gly, Ser and Thr is highest in violet Maca, as well as organic acids including butanoic acid and malic acid. Especially, the highest content of alanine and β-alanine, which is reported to regulate muscle carnosine synthesis [35], in violet Maca indicates the special function of improving the exercise performance of it. 

Conversely, some compounds have significantly higher content in one ecotype and might be used as marker compounds to identify Maca ecotypes—Mannose, 10,12-docosadiynedioic acid and hexanoic acid of black Maca, lactic acid, pipecolic acid and 2-pyrrolidinone of yellow Maca and β-alanine, butanoic acid and malic acid of violet Maca for example. Efficient marker compounds could be selected by screening in more Maca ecotypes. 

### 4.2. The Trancriptome of Maca

The authors obtained 115,866 unigenes of Maca hypotocyl and 63.10% of them were annotated. GO, COG and KEGG analysis indicated that the transport and metabolism of amino acids and carbohydrates were actively occurring in Maca hypotocyls, which confirmed it as a nutrient rich organ. Additionally, searching Maca unigenes against the transcriptome data of other Cruciferae members showed that the unigene sequences of Maca has a relatively high similarity to the genome data of *Arabidopsis thaliana*, *Arabidopsis lyrata* and another tuber root plant *Brassica rapa*, respectively (data not shown). The present genome information of Maca will facilitate the further studies on the regulatory mechanism of tuber root formation in the cruciferae family.

### 4.3. Different Expression Profiles and Specially Expressed Genes among Maca Ecotypes

The Kyoto Encyclopedia of Genes and Genomes analysis of DEGs showed that pathways of starch and sucrose metabolism, phenylpropanoid biosynthesis and phenylalanine metabolism include the largest number of DEGs. It is consistent with the results from metabolome data that Maca ecotypes have distinct profiles of the derivates of starch and sucrose metabolism. Phenylpropanoid biosynthesis is a key process for the biosynthesis of an enormous array of secondary metabolites, such as coumarins, tannins, lignin and flavonoids [36]. Phenylalanine is the initial compound of both phenylpropanoid and glucosinolate biosynthesis, therefore genes involved in these pathways might play important roles in determining the different content of essential active substances such as benzyl glucosinolate in Maca ecotypes. These DEGs linked to phenylpropanoid metabolism might led to the difference in glucosinolates among Maca ecotypes. However, the glucosinolates content in different Maca ecotypes required further measurement.

It is remarkable that DEGs between black and yellow Maca enriched in the pathway of “plant-pathogen interaction,” and a lot of “response to disease” and “disease resistant” genes were identified as DEGs between black and yellow Maca, indicating that the two ecotypes have different sensitivity or resistance to biotic stress. Combining with the fact that yellow Maca is the most widely planted ecotype with the largest yield, while black Maca is lowest in yield and demand to cultivate [5], it implied that the different expression of disease-related genes determines the viability of Maca ecotypes.

The authors discovered some genes especially expressed in one or two Maca ecotypes, which, at least partially, represent certain properties of the ecotype/s. The DNA ligase genes only expressed in black Maca suggest the occurrence of DNA damage and repair events [37]. The gene encoding disease resistance-responsive, dirigent domain-containing protein specially expressed in yellow Maca might play a role in disease resistance. A gene encoding chalcone synthase and a gene encoding MYB75 transcription factor were especially expressed in violet Maca and they are expected to play important roles in the accumulation of the violet color [38,39]. Consequently, a gene encoding ultraviolet-B receptor UVR8 was detected in black and yellow but not violet Maca, which might influence the UV-B signaling pathway in violet Maca. Additionally, these especially expressed genes are also potential molecular markers for the identification of Maca ecotypes.

The authors conducted a GC-MS-based metabolome analysis and de novo transcriptome sequencing of hypocotyls of Maca ecotypes. It was verified that different Maca ecotypes have distinct metabolic compositions and expression profiles. Yellow Maca is rich in carbohydrates and unsaturated fatty acid, black Maca is rich in protein and glucosinolate and violet Maca showed better antioxidant capacity. DEG analysis suggested that Yellow Maca have stronger resistance to disease, which might be a critical reason for its wide cultivation. MYB and WRKY transcription factors might play important roles in chemical composition determination of Maca. Genes encoding DNA ligase, ultraviolet-B receptor and chalcone synthase, as well as metabolites such as mannose, lactic acid and β-alanine, are candidate markers for Maca identification. While this study is a preliminary investigation on the chemical composition difference and underlying transcriptional regulation in Maca ecotypes, the further work to decipher the difference among ecotypes needs more experimental replicates for minimization of environment impacts. In addition, the measurement of anthocyanin content would be beneficial to directly distinguish the Maca ecotypes in the future. Overall, the genome resources generated in the present study will be valuable for further genetic and molecular investigations of Maca and lay the foundation for the use of modern molecular methods in efforts to improve the development and utilization of this species.

## Figures and Tables

**Table 1:** Functional annotation of Maca hypocotyl transcriptome.

Annotated Databases	Unigenes	≥300 nt	≥1000 nt
Nr	71,550	59,091	28,515
COG	49,846	42,993	24,192
KEGG	8287	8117	6133
GO	57,433	48,655	24,362
Swiss-Prot	51,936	43,669	23,091
All	73,113	59,614	28,548

**Table 2:** Specially expressed genes in Maca ecotypes.

Gene ID	Specially Expressed In ^1^	Swissprot Annotation	GO Annotation
c29661_g1_i1	B	Unknown	-
c34272_g1_i2	B	Unknown	-
c36979_g1_i2	B	No vein-like protein	-
c43742_g2_i1	B	Plastidic glucose transporter 4	-
c43908_g1_i6	B	DNA ligase	DNA recombination (GO:0006310)
c34364_g1_i4	Y	Glycine-rich RNA-binding protein	Cell wall (GO:0005618)
c36217_g1_i3	Y	Disease resistance-responsive, dirigent domain-containing protein	Defense response (GO:0006952)
c39509_g4_i3	Y	Zinc ion binding	-
c42330_g1_i1	Y	Probable fructokinase-5	Ribokinase activity (GO:0004747)
c51221_g3_i1	Y	Transposon Ty3-I Gag-Pol polyprotein	-
c34798_g1_i3	V	Unknown	Microtubule-based movement (GO:0007018)
c36120_g1_i1	V	Retrovirus-related Pol polyprotein from transposon TNT 1-94	-
-c40810_g1_i1	V	Chalcone synthase	Oxidation-reduction process (GO:0055114)
c67803_g1_i1	V	Unknown	-
c8503_g1_i1	V	Transcription factor MYB75	DNA binding transcription factor activity (GO:0003700)
c41540_g2_i3	BV	Ubiquitin-conjugating enzyme E2 8	Proteasome-mediated ubiquitin-dependent protein catabolic process (GO:0043161)
c41853_g5_i1	BV	Unknown	-
c42101_g1_i7	BV	Annexin D8	Response to water deprivation (GO:0009414)
c47327_g1_i3	BV	Unknown	-
c48799_g1_i1	BV	Aspartic proteinase-like protein 1	Anchored component of membrane (GO:0031225)
c13374_g1_i1	BY	Unknown	-
c20825_g2_i1	BY	Unknown	-
c21560_g1_i1	BY	Early light-induced protein 1, chloroplastic	Cytoplasm (GO:0005737)
c33991_g1_i2	BY	Unknown	-
c46340_g2_i8	BY	Ultraviolet-B receptor UVR8	Nucleotide-excision repair (GO:0006289)
c36101_g2_i1	YV	1-acylglycerol-3-phosphate O-acyltransferase	-
c36695_g1_i1	YV	Retrovirus-related Pol polyprotein from transposon TNT 1-94	-
c40657_g2_i3	YV	Thioredoxin O1, mitochondrial	Brassinosteroid biosynthetic process (GO:0016132)
c43332_g1_i8	YV	Tetratricopeptide repeat domain-containing protein	Response to sucrose (GO:0009744)
c45635_g1_i2	YV	Haloacid dehalogenase-like hydrolase domain-containing protein 3	NADP + binding (GO:0070401)
c49680_g2_i2	YV	Retrovirus-related Pol polyprotein from transposon TNT 1-94	Golgi apparatus (GO:0005794)

^1^ B, Y AND V represents black, yellow and violet Maca, respectively. “-” represents no match annotations in GO analysis.

## Supplementary Materials

The following are available online at http://www.mdpi.com/2073-4425/9/7/335/s1, Figure S1: Length distribution of the assembled unigene sequences, Figure S2: Functional annotation of assembled sequences based on gene ontology (GO) categorization, Figure S3: Clusters of orthologous groups (COG) classification, Figure S4: DEGs enriched in KEGG pathways, Figure S5: Expression patterns of genes involved in different pathways, Figure S6: Expression analysis by qPCR, Table S1: Summary of transcriptome data of Maca hypocotyls, Table S2: Summary of clean reads mapping into unigenes of 6 Maca samples, Table S3: Top 30 KEGG pathways contain largest number of enriched genes, Table S4: Primer Sequences used in qPCR analysis. Files A1–A7: Illumina sequence reads (also submitted to the NCBI Short Archive (SRA) under the BioProject PRJNA301501 (http://www.ncbi.nlm.nih.gov/bioproject/PRJNA301501)).
