# Supplementary material for: Combining Targeted Metabolites Analysis and Transcriptomics to Reveal Chemical Composition Difference and Underlying Transcriptional Regulation in Maca (Lepidium Meyenii Walp.) Ecotypes
Source: Genes (Basel). 2018 Jul 3;9(7):335. doi: 10.3390/genes9070335 (PMC6071217; doi:10.3390/genes9070335)
Supplement: Supplementary file 1 [file genes-09-00335-s001.zip › Supplementary files - revision/Supplementary Figures.doc]

**Figure S1. Length distribution of the assembled unigene sequences.**

Numbers in blankets represent the lengths of unigenes.

**Figure S2. Functional annotation of assembled sequences based on gene ontology (GO) categorization.**

The unigenes are summarized into three main categories: cellular component, molecular function and biological process.

**Figure S3. Clusters of orthologous groups (COG) classification.**

In total, 49,846 of the 115,866 sequences with Nr hits were grouped into 24 classifications.

black Maca vs. yellow Maca

black Maca vs. violet Maca

yellow Maca vs. violet Maca

**Figure S4. DEGs enriched in KEGG pathways.**

“Starch and sucrose metabolism”, “phenylpropanoid biosynthesis” and “phenylalanine metabolism” were identified the pathways contain largest enriched DEGs.

**A**

**B**

**Figure S5. Expression patterns of genes involved in different pathways.**

Genes involved in **A,** starch and sucrose metabolism and **B,** phenylalanine metabolism**,** phenylpropanoid biosynthesis and flavonoid biosynthesis are separately analyzed. The numbers of transcripts are indicated at the side of each step. The sample names are showed at the bottom.

**A**

**B**

**Figure S6. Expression anlysis by qPCR**

**(A)** Relative expression of 6 selected DEGs analyzed by qPCR. (**B)** Correlation of gene expression results obtained from qPCR and RNA-Seq.
