# Supplementary material for: Combining Targeted Metabolites Analysis and Transcriptomics to Reveal Chemical Composition Difference and Underlying Transcriptional Regulation in Maca (Lepidium Meyenii Walp.) Ecotypes
Source: Genes (Basel). 2018 Jul 3;9(7):335. doi: 10.3390/genes9070335 (PMC6071217; doi:10.3390/genes9070335)
Supplement: Supplementary file 1 [file genes-09-00335-s001.zip › Supplementary files - revision/Supplementary tables.doc]

**Table S1. Summary of transcriptome data of Maca hypocotyls**

Length represents the average length of the reads; Q20 represent the probability of a correct base call is 99%; Q30 represent the probability of a correct base call is 99.9%; N represents the ratio of undetermined bases in per million bases. b1 and b2, black Maca; y1 and y2, yellow Maca; v1 and v2, violet Maca.

| Samples | Length | Reads | Bases | Q20 (%) | Q30 (%) | GC (%) | N (ppm) |
| --- | --- | --- | --- | --- | --- | --- | --- |
| b1 | 122.73 | 30,848,678 | 3,786,198,076 | 99.20 | 97.78 | 44.48 | 6.40 |
| b2 | 122.84 | 45,781,430 | 5,623,970,401 | 99.24 | 97.89 | 44.05 | 1.77 |
| y1 | 122.88 | 40,438,218 | 4,969,184,322 | 99.30 | 98.04 | 44.07 | 1.84 |
| y2 | 122.68 | 51,381,664 | 6,303,436,359 | 99.31 | 98.08 | 44.10 | 1.83 |
| v1 | 117.71 | 42,848,426 | 5,043,730,967 | 97.99 | 94.49 | 43.88 | 1.36 |
| v2 | 117.63 | 43,583,118 | 5,126,588,319 | 97.98 | 94.46 | 43.97 | 1.39 |

**Table S2. Summary of clean reads mapping into unigenes of 6 Maca samples**

| Samples | Total reads | Total mapped | Unique mapped | Multi mapped | Mapped (%) | Unique (%) |
| --- | --- | --- | --- | --- | --- | --- |
| b1 | 30,848,678 | 20,130,848 | 12,968,512 | 7,162,336 | 65.26 | 42.04 |
| b2 | 45,781,430 | 29,886,450 | 19,335,800 | 10,550,650 | 65.28 | 42.24 |
| y1 | 40,438,218 | 26,386,922 | 16,893,794 | 9,493,128 | 65.25 | 41.78 |
| y2 | 51,381,664 | 32,875,022 | 21,068,754 | 11,806,268 | 63.98 | 41.00 |
| v1 | 42,848,426 | 28,666,712 | 18,761,766 | 9,904,946 | 66.90 | 43.79 |
| v2 | 43,583,118 | 29,167,090 | 18,928,730 | 10,238,360 | 66.92 | 43.43 |

| Pathway | Gene number |
| --- | --- |
| ko03010:Ribosome | 447 |
| ko01200:Carbon metabolism | 261 |
| ko01230:Biosynthesis of amino acids | 250 |
| ko03040:Spliceosome | 246 |
| ko04141:Protein processing in endoplasmic reticulum | 227 |
| ko04075:Plant hormone signal transduction | 206 |
| ko03013:RNA transport | 199 |
| ko00230:Purine metabolism | 183 |
| ko00190:Oxidative phosphorylation | 173 |
| ko05016:Huntington's disease | 169 |
| ko04120:Ubiquitin mediated proteolysis | 165 |
| ko00500:Starch and sucrose metabolism | 144 |
| ko05010:Alzheimer's disease | 138 |
| ko03015:mRNA surveillance pathway | 137 |
| ko03018:RNA degradation | 136 |
| ko05166:HTLV-I infection | 135 |
| ko04144:Endocytosis | 127 |
| ko05012:Parkinson's disease | 127 |
| ko00240:Pyrimidine metabolism | 126 |
| ko05169:Epstein-Barr virus infection | 124 |
| ko04110:Cell cycle | 124 |
| ko04111:Cell cycle - yeast | 121 |
| ko05203:Viral carcinogenesis | 120 |
| ko00010:Glycolysis / Gluconeogenesis | 117 |
| ko00520:Amino sugar and nucleotide sugar metabolism | 112 |
| ko03008:Ribosome biogenesis in eukaryotes | 112 |
| ko00564:Glycerophospholipid metabolism | 110 |
| ko04626:Plant-pathogen interaction | 108 |
| ko04114:Oocyte meiosis | 108 |

**Table S3. Top 30 KEGG pathways contain largest number of enriched genes**

**Table S4. Primer Sequences used in qPCR analysis**

| Primers | Primer sequences (5' to 3') | Product length（bp） |
| --- | --- | --- |
| c17076-g1-i1 forward | TCTTGACATCATTTGCGGTTCG | 189 |
| c17076-g1-i1 reverse | GAGCTGGGCTAAATCGGTGC |  |
| c21560-g1-i1 forward | CGACCAAACCGAGCATAGC | 101 |
| c21560-g1-i1 reverse | GCCACTTTTCAAGGGCGTAA |  |
| c35506-g1-i2 forward | TGACGCTACTGTTTTGTTACATCCT | 195 |
| c35506-g1-i2 reverse | CCATGTTTCACTTTTGGGAATATC |  |
| c40541-g1-i1 forward | ACGCCCATTGGCTCTGTTT | 243 |
| c40541-g1-i1 reverse | ATCGCTAAAGGGAAGGCACAT |  |
| c40810-g1-i1 forward | ACTTAGGAACTTCAACCACCACG | 104 |
| c40810-g1-i1 reverse | GCATCTGACGGAGGAGTTTCTT |  |
| c43742-g2-i1 forward | TTGAGGACGAAGGGGCACAG | 161 |
| c43742-g2-i1 reverse | TCACGCCACAAAGGGGAAGA |  |
| *LmActin* forward | TCAAGACGAAGAATAGCATGTGG | 203 |
| *LmActin* reverse | TCAAGACGAAGAATAGCATGTGG |  |
